# Focused Ultrasound, an Emerging Tool for Atherosclerosis Treatment: A Comprehensive Review

**DOI:** 10.3390/life13081783

**Published:** 2023-08-21

**Authors:** Cynthia Imtiaz, Muhammad Awais Farooqi, Theophilus Bhatti, Jooho Lee, Ramsha Moin, Chul Ung Kang, Hafiz Muhammad Umer Farooqi

**Affiliations:** 1 Ocean and Biomedical Ultrasound Laboratory, Department of Ocean System Engineering, Jeju National University, Jeju-si 63243, Republic of Korea; cynthiaimtiaz@stu.jejunu.ac.kr (C.I.);; 2 Department of Mechatronics Engineering, Jeju National University, Jeju-si 63243, Republic of Korea; 3 Interdisciplinary Department of Advanced Convergence Technology and Science, College of Pharmacy, Jeju National University, Jeju 63243, Republic of Korea; 4 Department of Pediatrics, Elaj Hospital, Gujranwala 52250, Pakistan; 5 Board of Governors Regenerative Medicine Institute, Cedars-Sinai Medical Center, Los Angeles, CA 90048, USA

**Keywords:** atherosclerosis, thrombolysis, focused ultrasound, high-intensity focused ultrasound, low-intensity focused ultrasound

## Abstract

Focused ultrasound (FUS) has emerged as a promising noninvasive therapeutic modality for treating atherosclerotic arterial disease. High-intensity focused ultrasound (HIFU), a noninvasive and precise modality that generates high temperatures at specific target sites within tissues, has shown promising results in reducing plaque burden and improving vascular function. While low-intensity focused ultrasound (LIFU) operates at lower energy levels, promoting mild hyperthermia and stimulating tissue repair processes. This review article provides an overview of the current state of HIFU and LIFU in treating atherosclerosis. It focuses primarily on the therapeutic potential of HIFU due to its higher penetration and ability to achieve atheroma disruption. The review summarizes findings from animal models and human trials, covering the effects of FUS on arterial plaque and arterial wall thrombolysis in carotid, coronary and peripheral arteries. This review also highlights the potential benefits of focused ultrasound, including its noninvasiveness, precise targeting, and real-time monitoring capabilities, making it an attractive approach for the treatment of atherosclerosis and emphasizes the need for further investigations to optimize FUS parameters and advance its clinical application in managing atherosclerotic arterial disease.

## 1. Introduction

Atherosclerosis is a prevalent chronic inflammatory condition that prompts plaques to accrue within the arteries and is the leading case of cardiovascular disease (CVD) [1]. According to the World Health Organization’s (WHO) global statistics, in 2019, an estimated 17.9 million people died from atherosclerosis-related complications, accounting for 32% of all global deaths. Atherosclerosis is a significant risk factor caused by the accumulation of cholesterol and fatty deposits in the arteries [2]. High blood pressure, diabetes, obesity, smoking, and a sedentary lifestyle can all substantially increase the risk [3]. Medical imaging is an effective method to screen and diagnose atherosclerosis [4,5,6,7]. Common treatments for this condition include lifestyle changes, medication, and surgery. Taking proactive steps to mitigate these risks is essential to prevent the onset of atherosclerosis and CVD [8]. 

The progression of atherosclerosis cause narrowing and stiffness of arteries, thereby reducing blood flow to vital organs and tissues [9]. Depending on the size of the artery blockage, the severity of symptoms can vary from mild discomfort to debilitating distress [10]. Atherosclerosis is a complex process that includes the progressive accretion of plaques within the arteries, causing the narrowing of the vascular lumen and consequent cardiovascular anomalies. A study revealed the association between radial artery and coronary calcification in adults with angina symptoms and associated risk factors [11]. Endothelial dysfunction or disruption to the interior layer of the artery walls is the root cause of atherosclerosis, and numerous accompanying risk factors such as smoking, high blood pressure, and excessive cholesterol levels can trigger this dysfunction [3,9,12]. Atherosclerosis-related diseases, such as coronary artery disease (CAD) and cerebrovascular disease, massively induce CVD mortality [10]. The progression of atherosclerosis might elicit the arteries to narrow and become stiffer, diminishing blood flow to vital tissues and organs. Given the size and enormity of the artery blockage, the symptoms could vary from mild discomfort to debilitating distress. Plaques accumulate due to an inflammatory response initiated by collecting fatty deposits, cholesterol, and other chemicals. These plaques can rupture, permitting clots to form that entirely prevent blood flow through the artery. Inflammation contributes to the progression of the disease by boosting plaque growth and weakening the artery walls. Understanding the effect of atherosclerosis underscores the magnitude of practical, proactive efforts, early identification, and proper treatment to mitigate its detrimental consequences [13]. Due to this malfunction, low-density lipoprotein (LDL) cholesterol may invade the artery wall. LDL cholesterol undergoes alterations once it infiltrates the artery wall. It is then metabolized by immune cells, primarily macrophages [14]. These immune cells differentiate into foam cells, the hallmark of initial plaques instigating atherosclerosis. In the artery wall, foam cells and other cells, lipids, and cellular debris produce fatty streaks. The fatty streaks develop into more complex plaques. Smooth muscle cells permeate the plaque from the artery wall, developing a fibrous cap [15]. The fibrous cap comprises collagen and other proteins and environs a lipid-rich core formed of foam cells, cholesterol, and cellular debris. As the plaques develop in all dimensions, the artery wall condenses and hardens, restricting the arterial lumen and dynamic blood flow. The narrowing of arteries can be ascribed to plaque depositions within the artery wall and the inflammatory reaction it causes [16]. 

**Figure 1:**
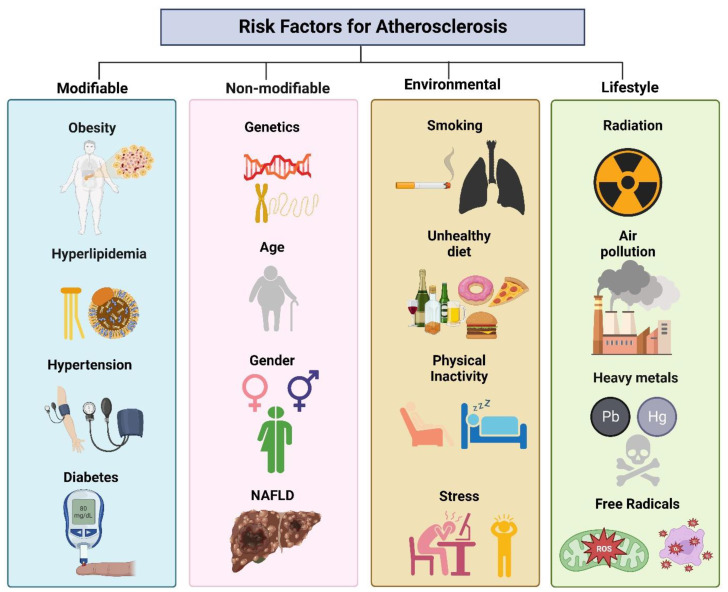
Association of risk factors linked with atherosclerosis.

Risk factors, including hypertension, smoking, diabetes, and dyslipidemia, can prompt atherosclerosis [17]. These risk factors are classified as modifiable and non-modifiable (Figure 1). Hypertension is a significant risk factor for developing atherosclerosis, and its chronic form detrimentally impacts the endothelial lining of the arteries, rendering them prone to lipid deposition and inflammatory cell expansion. Hypertension upsurges the strain on the heart, leading the artery walls to thicken and stiffen. Tobacco smoke incorporates chemicals that induce endothelial dysfunction, augment inflammation, and lead to fatty plaque formation inside artery walls. Smoking also lowers high-density lipoprotein (HDL) cholesterol levels, the “good” cholesterol, which assists in normalizing the overall blood cholesterol level [18]. Hyperlipidemia can cause elevated blood glucose levels, leading to diabetes and hasten plaque formation and endothelial damage [19]. Men are more inclined than premenopausal [20] to suffer from atherosclerosis. However, the risk juxtaposes in postmenopausal women [21,22]. Notably, these risk factors often interact and worsen the other’s consequences. These factors, including obesity, hyperlipidemia, hypertension, and diabetes, can be managed and controlled by lifestyle changes, regular medical check-ups, and medications that can reduce the clinical consequences [23]. In contrast, there are also non-modifiable factors of atherosclerosis etiology, i.e., genetics, gender, chronic nonalcoholic fatty liver disease (NAFLD), habitat, and environmental factors.

**Figure 2:**
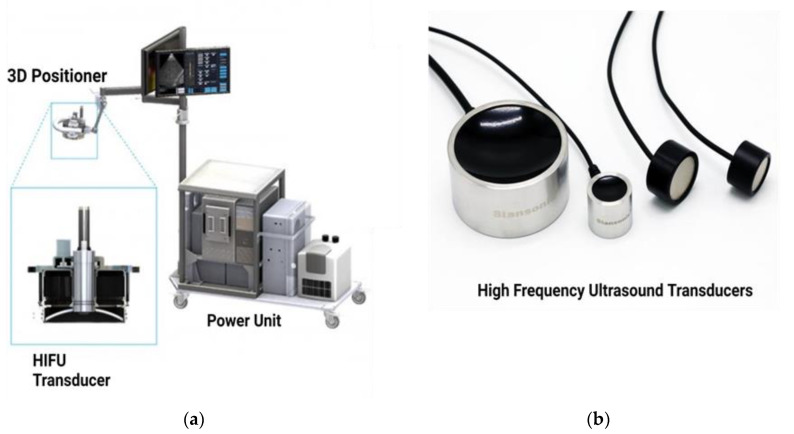
(**a**) FUS equipment. (**b**) HIFU transducers of varying sizes.

HIFU can assist in evaluating the stages of atherosclerosis, including its potential to mitigate plaque burden, promote vascular remodeling, and reduce adverse effects compared to conventional methods. It offers targeted and precise therapeutic benefits without invasive interventions. HIFU provides high spatial resolution, enabling accurate targeting of atherosclerotic plaques without damaging surrounding healthy tissues. The ability to focus the ultrasound beam on specific locations enhances the effectiveness of the treatment and reduces the potential for collateral damage [24]. HIFU procedure is associated with minimal downtime and a faster recovery than invasive surgeries. By potentially reducing plaque burden and improving vascular function, HIFU may decrease reliance on long-term medication, mitigating the risk of contradictory effects and improving patient compliance. However, HIFU technology needs optimization for treatment protocols, addressing potential safety concerns, and conducting large-scale clinical trials to establish long-term efficacy, safety, and clinical validation. Regarding HIFU equipment, two primary components must be taken into consideration. The first component is the piezoelectric ultrasound transducer that targets the therapeutic ultrasound beam, as shown in Figure 2. Although the most utilized transducer is a concave focusing transducer with a fixed aperture and focal length, other types of transducers are available, such as phased array transducers and flat transducers [25]. The transducer’s mechanical movement determines the focal point’s position, while electronic steering offers precise control of the focal spot location. The second significant component of HIFU is the imaging modality utilized for guidance. Real-time imaging is crucial during the therapeutic procedure to ensure the safety and efficacy of treatment. Sonography and Magnetic Resonance Imaging (MRI) are two imaging modalities used for monitoring treatment [24]. Figures displaying the schematics of typical ultrasound and MRI-guided focused ultrasound systems are applied to the target through the skin for extracorporeal shock wave therapy (ESWT) and HIFU.

There has been an emergence of HIFU as a potential noninvasive therapeutic modality for treating atherosclerotic arterial disease in various arterial districts. The coronary, carotid, and peripheral arteries are the most accessible arterial districts for FUS application [26]. Patients will lie prone on the procedure table when undergoing FUS for the femoral artery. Real-time ultrasound or MRI imaging guides the focused ultrasound waves to the targeted location within the artery. The transducer, equipped with a cooling system, is placed on the skin over the femoral artery, allowing the ultrasound energy to target the atherosclerotic plaque precisely. This leads to localized thermal ablation and mechanical disruption of the plaque, which promotes its breakdown and eventual resorption by the body [27]. A similar procedure is followed for the carotid artery, with the patient positioned supine or slightly inclined. FUS aims to reduce plaque burden and improve blood flow to the brain, minimizing the risk of stroke or other cerebrovascular events. The cooling system ensures that the energy delivery is precisely controlled, avoiding damage to the surrounding structures [28].

## 2. Current Treatment Options for Atherosclerosis

Antiplatelet medications, i.e., aspirin and clopidogrel, are often recommended to reduce the risk of blood clot formation and prevent repercussions. Antihypertensive drugs may be recommended if a patient has hypertension to manage blood pressure and reduce the strain on the artery walls. Proper glucose management through conventional medications, insulin, and lifestyle changes is critical in reducing the progression of atherosclerosis in diabetics [16]. In severe cases of atherosclerosis, intrusive operations may be required to restore blood flow and control magnitudes. Angioplasty, for instance, involves inflating a balloon within the restricted artery to broaden it and improve blood flow [29]. A stent (a tiny mesh tube) may be implanted in certain situations to maintain an artery canalized. Coronary Artery Bypass Grafting (CABG) is a surgical treatment that involves employing blood vessels retrieved from other body regions to bypass blocked or constricted coronary arteries. It is usually performed on people who have severe coronary artery disease. Carotid Endarterectomy is a surgical procedure that eradicates plaques from the carotid arteries and thus re-canalizes blood to the brain [30]. It lessens stroke risk for individuals with significant carotid artery stenosis.

**Figure 3:**
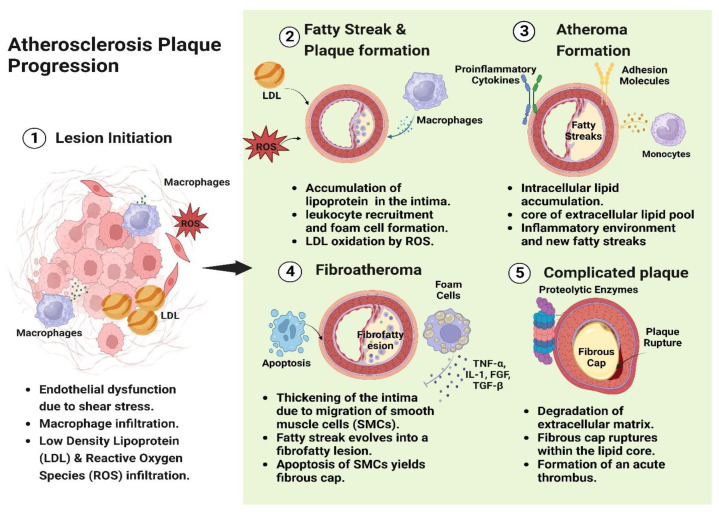
Characterization of atherosclerosis plaque progression from lesion initiation to formation of an acute thrombus.

The treatment designated varies by the severity and site of atherosclerosis, the individual’s general safety, and other specific factors. Figure 3 depicts the progression of atherosclerosis plaque from lesion initiation to complicated plaque and the time points that can be exploited for atherosclerosis prevention or treatment. The atherosclerotic pathogenesis starts with lesion instigation and occurs due to certain factors, mainly endothelial dysfunction and infiltration of lipoprotein and reactive oxygen species [14]. Then it begins into a fatty streak and plaque formation triggered by lipoprotein growth in the intima. Thirdly it progresses to atheroma formation, in which intracellular lipid is accumulated [31]. The next stage is the thickening of the intima evolves into a fibrofatty lesion called fibroatheroma. The last stage is the degradation of the extracellular matrix into an acute thrombus. Treatment strategies are often individualized to each patient’s needs to manage atherosclerosis and restrict the risk of problems. They may include lifestyle changes, drugs, and invasive procedures. Regularly, healthcare professionals must assess progress and modify the treatment plan [32,33].

## 3. Comparison of Invasive and Noninvasive Treatment Options for Atherosclerosis

**Figure 4:**
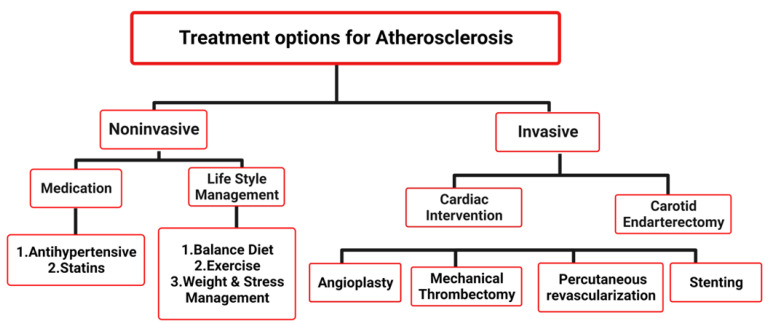
Invasive and non-invasive treatment options for atherosclerosis.

When it comes to the treatment of atherosclerosis, there are two approaches, invasive and noninvasive. Angioplasty, stenting, CABG), and carotid endarterectomy are invasive treatments that require physical intervention within the body. In Figure 4, specific surgical interventions and invasive and non-invasive treatment options have been outlined. Other invasive treatments for arterial blockages may involve thrombectomy, which removes blood clots from the arteries. This procedure can be performed through various methods, including catheter-based mechanical thrombectomy, surgical thrombectomy, and thrombolysis [3,28,34]. Catheter-based mechanical thrombectomy utilizes specialized catheters with mechanical devices or suction to physically disrupt and eliminate the clot from the artery, promoting blood flow restoration. Catheter-based laser atherectomy involves using a laser-equipped catheter to emit high-energy light, which vaporizes or removes the plaque, permitting improved blood flow through the artery. Rotational atherectomy is a procedure that exploits a specialized catheter with a rotating burr at its tip [29]. The burr shaves off and removes the plaque, opening the artery lumen and restoring blood flow. This technique is instrumental in cases where the plaque is calcified and resistant to other treatments [25]. These procedures are mentioned in Table 1 and have typically proven effective at restoring blood flow, alleviating symptoms, and reducing the risk of atherosclerosis-related complications. It nevertheless entails hazards that accompany invasive procedures, including bleeding, infection, blood clots, and an extended time for recovery [35]. Patients having advanced or severe coronary artery disease tend to be treated with invasive treatments. Noninvasive treatments do not involve surgery or physical intervention within the body. Instead, it relies on exogenous ways of targeting atherosclerosis and countering its risk factors [25]. Noninvasive therapies traditionally emphasize changes in behavior and drugs. Adopting a healthy diet, regular exercise, smoking cessation, and weight management are essential in preventing and controlling atherosclerosis. Medications such as statins, antiplatelet drugs, and antihypertensive are frequently utilized to manage cardiovascular risk factors and slow the advancement of atherosclerosis [29]. Noninvasive treatments are usually indicated for people with moderate forms of atherosclerosis or are unsuitable candidates for invasive procedures. While invasive treatments have proven efficacy, there is a need for innovative noninvasive approaches in the management of atherosclerosis [25]. Noninvasive treatment options offer several advantages, including reduced risk, shorter recovery time, and potentially lower healthcare costs. They are also more accessible to a broader range of patients and can be used as preventive measures in individuals at high risk of developing atherosclerosis. Focused ultrasound is a promising emerging field that holds potential for the noninvasive treatment of atherosclerosis [35]. It utilizes high-frequency sound waves to deliver precise energy to targeted tissues deep within the body without surgical incisions [36].

## 4. Focused Ultrasound as an Emerging Tool for Atherosclerosis Treatment

FUS has been approved as a noninvasive treatment option for various medical conditions. This innovative modality provides highly effective therapeutic benefits and is particularly effective in treating essential tremors and ablating prostate, hepatic, breast, and uterine tumors. Using a combination of ultrasound transducers with varying shapes, frequencies, and imaging systems, FUS delivers controlled energy to the target tissue with high precision and accuracy. Patients are positioned in either a prone or supine position during the procedure, and the ultrasound waves are directed at the target site through the skin, eliminating the need for invasive incisions [40]. The potential of FUS for treating atherosclerotic arterial disease is increasingly supported by a growing body of evidence, despite pending regulatory approval. Its effectiveness has been demonstrated in various medical fields, and noninvasive delivery of focused ultrasound waves to peripheral arteries such as the femoral, carotid, and iliac arteries is feasible. This targeted approach holds great promise for reducing atherosclerotic plaque burden and improving vascular function and deserves further exploration [28].

Currently, percutaneous endovascular techniques remain the predominant approach for treating arterial pathology. However, FUS offers a noninvasive alternative that holds promise as a potential adjunct or standalone therapy for atherosclerotic arterial disease. This review thoroughly examines the current evidence and scientific literature on using noninvasive FUS for atherosclerosis treatment. Our goal is to provide a clear understanding of FUS’s potential impact on revolutionizing the management of atherosclerotic arterial disease and shaping the future of noninvasive therapeutic interventions in this field [30]. This remarkable technology has given rise to two well-recognized techniques: HIFU and LIFU. HIFU utilizes highly focused ultrasound waves to generate intense heat within specific tissues, resulting in thermal ablation and tissue dissolution [41]. It has succeeded in noninvasive tumor ablation, offering an alternative to surgical removal. HIFU has effectively treated various solid tumors, such as tumors of the prostate, liver, kidney, breast, and uterus [42]. Real-time imaging guidance is often employed during HIFU procedures to ensure accurate targeting and allow healthcare professionals to monitor the fate of the treatment [43].

HIFU has also demonstrated promise in other clinical applications, such as treating essential tremors and certain neurological disorders. LIFU, on the other hand, utilizes focused ultrasound waves of lower intensities that are not associated with thermal ablation. Despite this, LIFU offers therapeutic benefits to various clinical conditions and pathologies [18]. It has gained recognition for its ability to stimulate or influence specific organs and metabolic processes. LIFU has shown potential in various therapeutic applications, including pain management, neuromodulation, neurostimulation, and targeted drug delivery. LIFU can target specific neural centers in pain management, providing localized pain relief without systemic medications. LIFU can influence neuronal activity in neuromodulation, potentially offering noninvasive treatments for conditions like Parkinson’s disease, Alzheimer’s disease, and epilepsy [40]. LIFU can also enhance drug administration by temporarily disrupting the blood-brain barrier, enabling drugs to reach the brain.

When it comes to HIFU, precision targeting has been a fundamental challenge. However, interesting technological advancements have allowed more accurate and effective treatments [44]. The U.S. Food and Drug Administration (FDA) has approved multiple clinical applications of HIFU, including uterine fibroid ablation, prostate cancer therapy, essential tremor treatment, and relief from bone metastasis discomfort [45]. The therapeutic effects of HIFU rely on two main physical mechanisms: thermal and nonthermal. By implementing higher intensities than those used in diagnostic ultrasound imaging, HIFU achieves its curative benefits [46]. Tissue ablation and coagulative necrosis occur by converting ultrasound energy into heat when tissues absorb the energy. Cavitation, the formation and fluctuation of microbubbles, enhances the nonthermal effects of HIFU. These effects include microstreaming, jetting, bubble expansion, compression, and stable or unstable cavitation, all contributing to the disruption of the target tissue [47].

HIFU transducers are designed to converge ultrasound beams at a focal point where localized biological effects occur to ensure precise targeting. Different beam-focusing methods, such as geometric focusing, acoustic lens focusing, and electronic focusing, are employed to achieve this [48]. Geometric focusing uses the shape of the transducer’s surface to concentrate the ultrasound waves [49]. In contrast, acoustic lens focusing involves employing an acoustic lens to simulate a concave surface transducer. On the other hand, electronic focusing utilizes phased array transducers with unique excitation signals to shift the focus point electronically without physically moving the transducer [50,51,52]. It enables accurate tissue targeting with minimal impact on the surrounding area. In addition to generating and focusing ultrasound beams, HIFU systems must be able to monitor the target tissue using magnetic resonance imaging or ultrasound examination in real-time. This allows for continuous imaging supervision during the treatment [37]. A real-time dual-mode ultrasound array (DMUA) system has been developed to address this need, enabling simultaneous imaging and therapy [53].

## 5. Overview of FUS Parameters for Achieving Sono Thrombolysis in Atherosclerosis Treatment

**a.**  **Intensity:**

Ultrasound intensity refers to the strength or energy of the ultrasound waves administered during treatment. It plays a critical role in the disruption of atherosclerotic plaques. Higher intensities result in more significant energy deposition, producing more pronounced mechanical and thermal effects on the plaques. The mechanical effects include acoustic streaming, microstreaming, and micro cavitation, which can mechanically disrupt the structure of the plaque [26]. The thermal effects involve localized heating, which can soften the plaque and enhance its susceptibility to mechanical disruption. However, finding the right equipment in intensity prevents adverse effects such as tissue damage or vascular complications. As mentioned in Table 2, different parameters have been evaluated by FUS. The appropriate level of intensity depends on various factors, including the size and composition of the plaque, patient-specific characteristics, and the specific ultrasound device employed [54].

**b.**  **Frequency:**

Ultrasound frequency refers to the rate at which ultrasound waves are generated per unit of time [49]. This parameter determines how ultrasound interacts with targeted tissues and the resulting therapeutic effects. Lower frequencies can penetrate deeper into tissues but may have limitations regarding spatial resolution [55]. On the other hand, higher frequencies offer better spatial resolution but have a shallower depth of penetration. The choice of frequency depends on the specific location and depth of the targeted atherosclerotic plaque. Lower frequencies are often suitable for treating deeper plaques within the body, such as those in larger arteries [46]. Conversely, higher frequencies can effectively target superficial plaques, such as those found in carotid arteries [41]. By selecting the appropriate frequency, optimal energy deposition can be achieved, enhancing the mechanical and thermal effects on the plaque. For instance, in Table 2, various studies utilized frequencies ranging from 1 MHz to 3.5 MHz for sonothrombolysis and plaque disruption, depending on their specific objectives and target tissues [43].

**c.**  **Duration of exposure:**

“Ultrasound exposure” refers to the duration of ultrasound waves applied during treatment. This parameter must be carefully regulated to achieve FUS-mediated plaque dissolution while avoiding harmful effects effectively [18]. Prolonged exposure can increase the likelihood of tissue heating and vessel damage. Therefore, it is necessary to find the right balance by selecting an adequate exposure duration to induce mechanical plaque disruption while minimizing the risk of complications [56]. The optimal duration of exposure can vary based on various factors, including specific treatment parameters, plaque characteristics, and individual patient considerations, e.g., different studies have utilized pulse lengths ranging from milliseconds to seconds, depending on their experimental design and treatment objectives [40].

**d.**  **Spatial targeting:**

Accurate treatment delivery in focused ultrasound (FUS) for atherosclerosis is paramount to curtail the destruction of healthy tissues and ensure precise treatment [39]. Accurate localization of the ultrasound beam on the atherosclerotic plaque is critical to concentrate the healing energy and optimize its impact on the desired area [28]. Real-time visualization of the plaque using imaging techniques like ultrasound or other modalities enables clinicians to focus the ultrasound beam precisely [57]. Imaging guidance allows clinicians to locate and target the plaque, guaranteeing energy delivery to the intended location. This precise spatial targeting enhances the effectiveness of sonothrombolysis while reducing the potential for complications or damage to neighboring structures [58].

## 6. Mechanisms of Focused Ultrasound Thrombolysis and Clinical Outcomes

Intravenous (IV) thrombolysis with a hybrid tissue plasminogen activator (rt-PA) is the recommended treatment for emergency thrombosis in stroke patients. This form of a thrombolytic agent, approved by the European Union and the FDA, has proven to be the most striking and validated intervention [59]. Administering rt-PA within 4.5 h of symptom onset can inherently recuperate the patient’s prognosis. However, the utilization of IV rt-PA remains subsided, with less than 5% of acute ischemic stroke (AIS) patients receiving this treatment. Furthermore, only 30–40% of patients achieve arterial recanalization, and even fewer, around 18%, achieve complete and sustained recanalization [60]. As a result, many individuals suffer severe brain damage, leading to high rates of disability and mortality. There is also a considerable risk of clinical subcortical hemorrhage associated with rt-PA therapy. Given the limitations of thrombolytic medication in achieving prompt arterial recanalization, it is necessary to explore novel treatment approaches that can complement recanalization [39].

**Figure 5:**
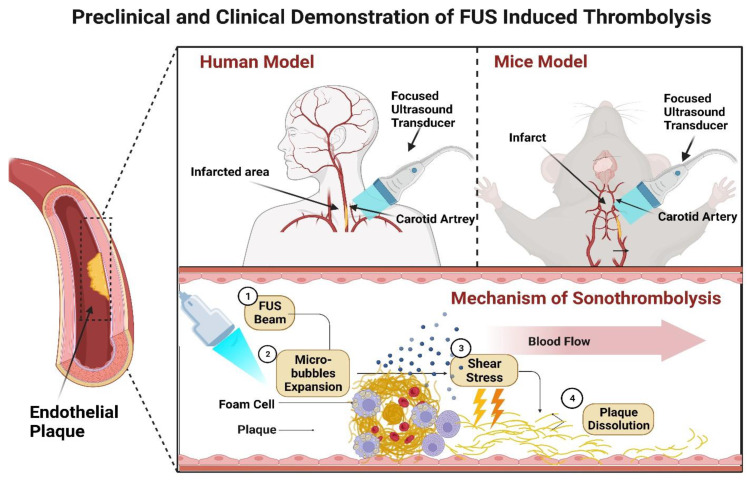
The preclinical and clinical demonstration of FUS-induced thrombolysis and the sonothrombolysis mechanism.

One remarkable advancement in this regard is incorporating ultrasound (US) energy to boost the therapeutic effects of thrombolytic drugs. Sonothrombolysis, or US-enhanced thrombolysis, is an assuring technique for treating AIS, as shown in Figure 5. It involves using transcranial US to augment the efficacy of thrombolytic medications [61]. Numerous in vitro and in vivo studies have endorsed that US energy can expedite thrombolytic therapy by elevating the catalytic impact of these drugs. Although the exact mechanisms underlying sonothrombolysis are not fully understood, US energy is postulated to facilitate fluid movement around the occlusion [23].

Additionally, the US undermines fibrin cross-links, allowing for increased uptake, infiltration, and concentration of rt-PA at the clot’s binding sites. Furthermore, the infusion of gaseous microbubbles (MBs), which act as US contrast agents, has shown potential in further enhancing US-enhanced rt-PA-induced thrombolysis [62]. When exposed to a US field, these MBs oscillate, expand, or collapse, resulting in either stable cavitation (microstreaming) or inertial cavitation (microjetting). Stable cavitation, characterized by sustained bubble activity, agitates the fluid surrounding the spheres, leading to increased distribution of thrombolytic. Inertial cavitation, on the other hand, involves a rapid increase in bubble size followed by a collapse, which can mechanically disintegrate the thrombus [59]. In recent years, FUS has emerged as a promising method for sonothrombolysis. This innovative approach facilitates the noninvasive treatment of clots by transmitting high-pressure ultrasound waves into millimeter-sized focal volumes through focused transducers.

The energy deposition outside the focal volume is minimal, ensuring no permanent damage to the surrounding tissue [63]. By optimizing the parameters of FUS through nonthermal mechanisms, the penetration and binding of the drug into the clot can be maximized, resulting in increased enzymatic thrombolysis. A review of studies and clinical trials showcasing the efficacy of FUS in treating is provided in Table 2. These studies have revealed that FUS treatment effectively disrupts and fragments atherosclerotic plaque, reducing plaque size and thickness. Additionally, FUS has been found to stimulate the release of natural thrombolytic agents and promote vascular remodeling, contributing to the restoration of vascular health [64]. A recent study used a dual-mode ultrasound array in a swine model of peripheral arterial disease (PAD). They demonstrated the disruption of atherosclerotic plaques without causing damage to the endothelial lining [10]. It suggests that FUS has the potential to selectively target and break down plaques, offering a promising strategy for mitigating the progression of atherosclerosis. A research group observed increased angiogenesis in the ischemic hind limbs of a rat model of PAD following FUS treatment. Angiogenesis, forming new blood vessels, is crucial in restoring blood flow and tissue perfusion. These findings indicate that FUS may enhance angiogenesis, improving vascular function and tissue healing in ischemic conditions [64].

Furthermore, some studies demonstrated FUS’s positive effects on perfusion and atherosclerosis reduction. In diabetic mice and rabbits, FUS treatment led to increased perfusion and a decrease in plaque burden, accompanied by changes in angiogenic factors, antiapoptotic factors, and cellular composition [65]. Table 2 overviews the preclinical and clinical data of HIFU in thrombotic arterial disease. These findings suggest that HIFU can improve blood flow and promote plaque regression, offering a novel approach to managing atherosclerotic disease. FUS has also shown promise in thrombolysis, with some previous studies demonstrating enhanced thrombolysis and partial restoration of blood flow in different models of blood clot formation [66]. The mechanisms underlying these effects involve increased uptake of tissue plasminogen activator (tPA) and cavitation, wherein microbubbles generated by FUS induce mechanical stress on the clot, facilitating its dissolution. These findings suggest that FUS could be used as an adjunct therapy to improve thrombolysis outcomes in patients with occlusive arterial diseases [67]. Targeted microbubbles combined with low-power focused ultrasound can reduce inflammation, significantly promote sonothrombolysis, and provide new ideas and methods for the diagnosis and treatment of acute DVT LIFU irradiation for coronary thrombolysis and PA-mediated clot dissolution verified its treatment effect with high-efficient FUS-mediated plaque dissolution in the in-vivo experiments, which can be considered as robust experimental evidence of the novel method for potential clinical use of sonothrombolysis [36,68].

Furthermore, one recent study used that vortex ultrasound causes no vessel wall damage over ex vivo canine veins. This vortex ultrasound thrombolysis technique potentially achieved recanalization of blood flow in cerebral venous sinus thrombosis (CVST) [48]. Another study investigated ultrasound-targeted microbubble cavitation during machine perfusion to reduce microvascular thrombi and graft injury in a rat liver donation model after circulatory death (DCD). The researchers found that this technique effectively reduced microvascular thrombi formation and minimized graft injury in the DCD liver model [65]. Using ultrasound and microbubbles to enhance perfusion and reduce thrombi formation shows a potential therapeutic approach for improving outcomes in DCD liver transplantation. While the studies mentioned earlier have provided valuable insights into the effectiveness of FUS in reducing plaque burden, promoting angiogenesis, and enhancing FUS-mediated plaque dissolution, further research is necessary to optimize FUS settings and develop precise image-guided techniques [13,71]. Additionally, clinical trials are needed to evaluate FUS’s safety, feasibility, and long-term effectiveness in human subjects. Nevertheless, the current findings in Table 2 highlight the potential of FUS as a noninvasive therapeutic approach that could revolutionize the treatment of atherosclerotic arterial disease.

## 7. Comparison of FUS with Traditional Treatment Methods

FUS offers significant advantages over standard therapy modalities, including invasive procedures and pharmaceutical therapies, regarding clinical outcomes [53,75]. One key advantage of FUS is its noninvasiveness, which eliminates the need for surgical incisions or the insertion of catheters or devices into the body [76]. It reduces the risk of clinical complications associated with invasive procedures, such as infections, bleeding, surgery-associated anxiety and depression, and scars. Furthermore, noninvasive treatment reduces the need for hospitalization, leading to shorter recovery periods and lower healthcare expenses [77]. Another benefit of FUS is its ability to provide focused therapy. Ultrasound energy can be precisely targeted to specific regions of interest, such as atherosclerotic plaques. It allows targeted treatment while preserving healthy surrounding tissues, as shown in Figure 4. The FUS transducer is only exposed to the infracted area of the carotid artery [35].

In contrast, invasive procedures may involve more extensive interventions that can affect both diseased and healthy tissues, potentially resulting in side effects or adverse effects. FUS also offers the advantage of customized therapy [25]. Parameters mentioned in Table 2, such as intensity, frequency, and duration of ultrasound, can be adjusted to meet each patient’s specific needs and targeted tissues [30,35]. This personalized approach ensures optimal treatment outcomes while minimizing side effects. In contrast, pharmacological therapies may have limited adaptation options due to individual drug metabolism variations [78].

Additionally, FUS enables real-time imaging, allowing medical professionals to monitor and track therapy progress. This immediate feedback enables adjustments during the treatment to ensure accurate targeting and enhance therapeutic effectiveness [72]. Invasive procedures may not always provide the same level of real-time imaging accessibility. In summary, FUS offers substantial advantages in clinical outcomes compared to standard therapies. Its noninvasive nature, focused therapy, customization options, and real-time imaging improve treatment efficacy and patient safety [79].

## 8. Merits of FUS in Atherosclerosis Treatment

FUS holds immense potential as a noninvasive therapeutic method in managing atherosclerosis, offering numerous resources that make it an imploring option for this complex arterial condition. One paramount profit of FUS is its noninvasive nature, waiving the requirement for surgical procedures and minimizing associated hazards and complications. Unlike conventional interventions like endovascular procedures or surgeries, FUS can precisely target atherosclerotic plaques without damaging proximal healthy tissue [80]. This targeted approach minimizes procedural hazards and accelerates patient recovery. Moreover, FUS provides a localized and specific treatment option for atherosclerosis. FUS can disrupt and break down atherosclerotic plaques by focusing high-intensity ultrasound waves on affected arterial segments [81]. This targeted energy helps recede plaque burden, sustain vulnerable plaques, and revitalize normal blood flow in diseased arteries. By addressing the underlying cause of atherosclerosis, FUS has the potential to halt or even reverse disease progression [82].

Additionally, FUS offers therapeutic benefits beyond plaque reduction. Studies have substantiated its capability to prompt angiogenesis, forming new blood vessels in ischemic tissues [83]. This angiogenic response can strengthen tissue perfusion and oxygenation, which is central for recovering and healing afflicted arteries. Furthermore, FUS allows real-time monitoring and evaluation throughout the procedure. Advanced imaging techniques like ultrasound or MRI can be integrated with FUS to provide precise guidance and monitoring throughout the treatment [31,50]. This legitimizes clinicians apprehending the targeted area, regulating treatment parameters, and ensuring optimal energy distribution to the desired area. In conclusion, FUS shows immense promise as a noninvasive and targeted therapeutic approach for atherosclerosis treatment. Its ability to selectively disrupt plaques, stimulate angiogenesis, and offer real-time monitoring make it a potentially pragmatic strategy for reducing plaque burden, vascular remodeling function, and revolutionizing the management of atherosclerotic arterial disease [14,47,49].

## 9. Limitations and Future Perspectives

The extensive utilization of HIFU in numerous disorders is hindered by several limitations, despite its effective use in achieving sonothrombolysis in clinical practice. One of these limitations is the challenge of tissue penetration, particularly in abdominal regions such as the aorta or iliac arteries. This hampers the treatment of deep vein thrombosis (DVT) and occluded vessels embedded deeply within body tissues. Another obstacle encountered by high-frequency focused ultrasound is the presence of gas or bone structures, which can cause reverberation and impair the adequate delivery of ultrasound waves, leading to suboptimal treatment outcomes [69]. In clinical trials, additional reflections of these ultrasonic waves can implicitly induce tissue damage and skin burns, and initiating conscientious patient selection is pivotal for ensuring treatment efficacy and safety. Factors such as the site and severity of atherosclerotic lesions and tissue-specific characteristics must be considered.

Moreover, HIFU, if not utilized properly, poses a risk for adverse side effects and potential harm to healthy endothelial cells, including tissue necrosis. High-intensity frequencies can induce inertial cavitation, which can cause scarring to vascular endothelial cells and elevate the risk of complications [57,58]. Inadequate acoustic coupling at the skin-transducer interface can also lead to skin burns. Another notable pitfall of currently available HIFU technology is the limited ability to sufficiently image, focus, target, and monitor tissues in real-time during treatment [70]. While a substantial body of research reinforces the employment of HIFU in atherosclerotic artery disease, further corroboration is required to validate its efficacy in certain conditions associated with promising outcomes. Recent advancements have been employed to cope with these challenges, such as using DMUA ultrasonic probes or magnetic resonance imaging-guided focused ultrasound (MRgFUS). MRgFUS proposes the advantage of real-time temperature monitoring; however, it is an expensive and resource-intensive approach due to the reliance on MRI-guided thermometry [50]. The second barrier to overcome is the need for a standardized process. A consistent methodology is obligatory to evaluate and rank FUS’s histologic, radiographic, and clinical impacts on atherosclerotic treatment. Several areas of future research can be explored to tackle the limitations of FUS treatment for atherosclerosis. One potential avenue is to characterize FUS parameters using tissue-mimicking phantoms or numerical simulations. By employing phantoms that mimic the acoustic properties of human tissues, researchers can gain insights into the effects of different FUS parameters, such as intensity, frequency, and exposure duration, in a controlled laboratory setting. This approach systematically evaluates parameter settings and their impact on the effectiveness and safety of FUS-mediated plaque dissolution. Additionally, numerical models can provide valuable predictions of the distribution of the acoustic field and temperature effects within the target tissue, aiding in optimizing treatment protocols [84].

Furthermore, including cavitation control mechanisms in transducer designs, such as feedback systems that monitor and regulate cavitation activity, can help prevent excessive or undesired effects on vascular endothelial cells and ensure safer and more successful treatment outcomes [85]. In addition to these research areas, a crucial future direction in FUS for atherosclerosis treatment involves the development of HIFU probes with tissue temperature monitoring capabilities [24]. Real-time temperature monitoring during FUS therapy offers valuable feedback and control over thermal effects, leading to optimal therapeutic outcomes while minimizing the risk of tissue damage [8,83]. Researchers can continuously monitor temperature changes within the target tissue during FUS treatment by integrating temperature sensors directly into the HIFU probe or by employing external monitoring techniques like infrared thermography or magnetic resonance thermometry [27]. This real-time feedback enables precise control of the thermal dose delivered to the atherosclerotic plaque, ensuring the desired therapeutic effects, such as sonothrombolysis, are achieved while avoiding excessive tissue heating and potential complications [86].

## 10. Conclusions

Employing FUS, particularly HIFU, a powerful noninvasive therapeutic approach, can potentially revolutionize the treatment of atherosclerotic artery disease. Studies stipulate that HIFU has the propensity to effectively stabilize susceptible plaques and decelerate the progression of atherosclerotic plaque development. By complementing medical therapy, HIFU renders a targeted treatment option for profuse arterial areas, leading to a reduction in plaque thickness and an enlargement of the vessel lumen, all while minimalizing the risks accompanying neointimal hyperplasia usually associated with current endovascular interventions. Future HIFU research will emphasize refining image-guided procedures and optimizing treatment parameters. Further investigation is imperative to establish the optimal HIFU settings that yield the desired biological effects.

## Figures and Tables

**Table 1:** Overview of high-intensity focused ultrasound (*HIFU*) clinical data in thrombotic arterial disease.

Setup	Study Pattern	Principle	Intensity (W/cm^2^)	Frequency	Clinical Relevance	Results	Reference
Ultrasound Angioplasty Ablation System	Invasive	Biomechanical	115	19.5 kHz	86% of lesions recanalized with ultrasound	Decreased arterial sclerosis	[25]
Dedicated ultrasound device	Noninvasive	Biomechanical	Individually calculated	1 MHz	Atorvastatin and Son dynamic therapy decreased diameter stenosis in PAD. lesions after four weeks	Decreased induration of the arteries	[37]
Sonos 5500	Noninvasive	Biomechanical	NA	1.3 MHz	Continuous The ultrasound did not affect perfusion in limbs, but ultrasound in PAD patients, it showed. microbubble cavitation increased perfusion	Lower the contradictory effects of ultrasound	[38]
CardioProlific Genesis System	Invasive	Biomechanical	NA	20 kHz	The treatment group has shown improvement. primary patency rates at six and 12 months	Improved efficacy of Sono thrombolysis	[24]
500M Transcranial Doppler System	Noninvasive	Biomechanical	128	2 MHz	Complete recanalization is achieved. in 36% of patients; clinical recovery achieved in 20% of patients	Peripheral arterial vasodilation	[26]
ATL Ultramark 9 HDI	Noninvasive	Biomechanical	415	2 MHz		Improved efficacy of Sono thrombolysis	[39]
PMD 100	Noninvasive	Biomechanical	750	2 MHz	Ultrasound and tPA administration resulted in complete recanalization clinical recovery from acute ischemic stroke	Increased arterial blood flow	[27]
TCD 100M	Noninvasive	Biomechanical	385	2 MHz	The type of microbubbles did not affect recanalization rates, clinical improvement, bleeding, in-hospital mortality, or long-term outcome	Decreased Contradictory effects	[1]
Dedicated cervical orthotic device.	Noninvasive	Biomechanical	0.75–1	800 kHz	Decreased thickness and area of carotid plaques	Decreased plaques	[1]

**Table 2:** Overview of high-intensity focused ultrasound (HIFU) clinical data in thrombotic arterial disease.

Model	Study Design	Duration of Exposure	Frequency	Duration of Exposure	Results	Ref.
Rabbit	Custom device with amplifier	10–40	1 MHz	Biomechanical	Decrease in carotid artery atherosclerosis through decreased neointima formation, macrophage	[37]
Adult human	Custom apparatus made with piezoelectric transducer	1	1 MHz	Biomechanical	Enhanced Sonothrombolysis via increased uptake of tPA	[15]
Adult human	Sonic Concepts	560–2360	1.1 MHz	Biomechanical	Cavitation and hemolysis is greater in samples with contrast agent treated with ultrasound	[34]
Adult human	Sonicator model XL 2020	NA	20 kHz	Biomechanical	Ultrasound and nongas-filled particles (HAEMACCEL and HAES) decreased clot burden	[46]
Rabbit	ExAblate 4000	66–200	220 kHz	Biomechanical	Mild recanalization in carotid artery stroke thrombosis model, dependent on platelet-activation and cavitation	[64]
Rabbit	Custom using function generator	300	1.51 MHz	Biomechanical	Increased sonothrombolysis and partial blood flow restoration in femoral artery	[69]
Rabbit	Custom device with amplifier	10–40	1 MHz	Biomechanical	Enhanced sonothrombolysis in rabbit carotid model via increased uptake of tPA	[28]
Adult human	Sonacell Multiphone	NA	0.75, 1.5., 3.0 MHz	Biomechanical	Release of bthromboglobulin in platelets is mediated by ultrasound induced cavitation	[15]
Swine	Sonic Concepts	2500–3100	3.5 MHz	Thermal	Control of arterial hemorrhage	[34]
Rabbit	Custom made. 111F-U applicator with piezoelectric discs	3000–6100	3.5 MHz	Thermal	Control of arterial hemorrhage	[70]
In vitro	Pulsed Doppler US	NA	5.7 MHz	Biomechanical	tPA and US-induced clot dissolution	[65]
DVT rabbit model	Low-Power Focused Ultrasound Device	2.0	0.7 MHz	Thermal	Low-power focused ultrasound reduces inflammation and promotes FUS-mediated plaque dissolution	[40]
N/A	Mathematical model	NA	1.1 MHz	Thermal	To predict the damage of plaque ablation based on wall thickness;	[71]
Swine	Imasonic Dual- Mode US Array	4100–5600	3.5 MHz	Thermal	Disruption of atherosclerosis in swine PAD model, accompanied by aggregates of lipid laden macrophages with necrosis.	[35]
(PAD) Sprague Dawley rat	Duolith SD1	0.1	1.054 MHz	Biomechanical	Increase in angiogenesis in hindlimb ischemia model for PAD	[46]
C57BL./6J mice (diabetic)	Custom transducer	0.3	1 MHz	Biomechanical	Increased perfusion in hindlimb ischemia model for PAD accompanied by increased angiogenic factors,	[72]
Mice (ApoEee)	Custom transducer	0.3	1 MHz	Biomechanical	Inhibition of atherosclerosis via reduction of LDL oxidation	[31]
Mice (ApoEee), Rabbit	Custom transducer	0.1–0.4	1 MHz	Biomechanical	Decrease in atherosclerosis in femoral arteries through decrease in macrophages and lipids	[26]
Swine	HIFU Synthesizer, International Cardio Corporation	1.5	1 MHz	Thermal	Targeting the dorsal wall of the external femoral artery without endothelial damage	[73]
Mice (C57BL6)	EPIQ 7	NA	3.5 MHz	Biomechanical	Increased perfusion in hindlimb ischemia model for PAD	[38]
Porcine model	XL2020, Sonic	6.0	1 MHz	Thermal	LIFU irradiation for coronarySono thrombolysis	[66]
(3D) Phantom flow model	vortex ultrasound transducer array	NA	1.8 MHz	Biomechanical	In vitro vortex sonothrombolysis in cerebral venous sinus thrombosis	[48]
(PAD) Sprague-Dawley rat	SONOS 7500, Philips	NA	1.3 MHz	Biomechanical	Decreases in hepatic arterial and portal venous flow resistance	[74]

## Data Availability

The data supporting this study are available from the corresponding author upon reasonable request.
